# Increased Aperiodic Neural Activity During Sleep in Major Depressive Disorder

**DOI:** 10.1016/j.bpsgos.2022.10.001

**Published:** 2022-10-25

**Authors:** Yevgenia Rosenblum, Leonore Bovy, Frederik D. Weber, Axel Steiger, Marcel Zeising, Martin Dresler

**Affiliations:** a Donders Institute for Brain, Cognition and Behavior, Radboud University Medical Center, Nijmegen, the Netherlands; b Department of Sleep and Cognition, Netherlands Institute for Neuroscience, Amsterdam, the Netherlands; c Max Planck Institute of Psychiatry, Munich, Germany; d Centre of Mental Health, Klinikum Ingolstadt, Ingolstadt, Germany

**Keywords:** Antidepressants, Aperiodic power, Excitation-to-inhibition ratio, Impaired sleep, Major depressive disorder, Neural noise

## Abstract

**Background:**

In major depressive disorder (MDD), patients often express subjective sleep complaints, while polysomnographic studies report only subtle alterations of the electroencephalographic signal. We hypothesize that differentiating the signal into its oscillatory and aperiodic components may bring new insights into our understanding of sleep abnormalities in MDD. Specifically, we investigated aperiodic neural activity during sleep and its relationships with sleep architecture, depression severity, and responsivity to antidepressant treatment.

**Methods:**

Polysomnography was recorded in 38 patients with MDD (in unmedicated and 7-day-medicated states) and 38 age-matched healthy control subjects (*N=* 76). The aperiodic power component was calculated using irregularly resampled auto-spectral analysis. Depression severity was assessed with the Hamilton Depression Rating Scale. We replicated the analysis using 2 independently collected datasets of medicated patients and control subjects (*N* *=* 60 and *N* *=* 80, respectively).

**Results:**

Unmedicated patients showed flatter aperiodic slopes compared with control subjects during non–rapid eye movement (non-REM) stage 2 sleep (*p =* .009). Medicated patients showed flatter aperiodic slopes compared with their earlier unmedicated state (*p* values < .001) and control subjects during all sleep stages (*p* values < .03). In medicated patients, flatter aperiodic slopes during non-REM sleep were linked to the higher proportion of N1, lower proportion of REM, delayed onset of N3 and REM, and shorter total sleep time.

**Conclusions:**

Flatter slopes of aperiodic electroencephalographic power may reflect noisier neural activity due to increased excitation-to-inhibition balance, representing a new disease-relevant feature of sleep in MDD.

Major depressive disorder (MDD) is a common psychiatric disorder characterized by at least 2 weeks of pervasive low mood, anhedonia, inappropriate guilt, and feelings of worthlessness (1). In 2017, MDD affected ∼2% of the world population (2). The percentage of people who are affected at some point in their life varies from 7% to 21%, reflecting the fact that MDD is a serious public health problem (2). Besides abnormalities of mood and affect, patients with MDD often have sleep complaints, including insomnia (in ∼60%) or hypersomnia (in ∼15%), as well as fatigue, excessive daytime sleepiness, and lack of concentration while awake (3). Broad evidence suggests that disturbances of sleep–wake rhythms and the circadian timekeeping system underlie the pathophysiology of depression (4). Understanding the mechanisms of these alterations might bring new insights into the understanding of MDD.

Intriguingly, whereas some polysomnographic studies have confirmed subjective sleep complaints of the patients by reporting decreased slow-wave and delta amplitudes, higher spindle amplitude, lower spindle density, and a more dispersed slow-wave-spindle coupling, others have suggested that oscillatory changes in MDD might be more subtle (5,6). One of the possible explanations for the divergent oscillatory findings is the confounding effect of aperiodic (i.e., non-oscillatory, scale-free) activity (7, 8, 9). For that reason, it has recently been recommended to differentiate the total electroencephalographic (EEG) spectral power into its oscillatory and aperiodic components in order to avoid misrepresentation and misinterpretation of the data while studying oscillations (7, 8, 9). In addition, exploring aperiodic activity is important per se, as it is a distinct type of brain dynamics with its own functional significance and rich information content able to provide a window into diverse neural processes (8, 9, 10).

Currently, aperiodic activity receives increasing attention with reports on aperiodic changes associated with sleep phases, tasks, age, and disease (7, 8, 9, 10, 11, 12, 13, 14, 15, 16). Notably, it has been shown that the slope of the aperiodic component reflects the ratio between excitatory and inhibitory currents in the brain (10,15,16), while the height of the spectra is related to neural spiking rates (8,10). Besides this, a steeper aperiodic spectrum can also reflect greater synchronization, while a flatter spectrum can indicate reduced synchronization (i.e., greater neural noise) (14). In view of the crucial role of the proper balance between neural excitation and inhibition (E/I) for healthy cognition, behavior (17), and sleep, aperiodic activity seems to be a promising tool for investigating MDD with its cholinergic, monoaminergic (18,19), glutamatergic (20), and GABAergic (gamma-aminobutyric acidergic) (21,22) imbalance. In MDD, the E/I ratio could be further affected by prescribed antidepressants.

In view of this background, here we explore aperiodic activity during sleep in MDD and its relationships with sleep architecture, depression severity, and responsivity to antidepressant treatment. This study has an exploratory nature with no a priori hypothesis on the direction of aperiodic changes.

## Methods and Materials

### Participants

We retrospectively analyzed polysomnographic recordings from a previous study conducted at the Max Planck Institute of Psychiatry, Munich, Germany (6). The sample consisted of 40 patients with MDD and 40 healthy control subjects individually matched by age (±2 years of tolerance) and gender (Table 1). None of the patients were treated with sedative antidepressants.

**Table 1:** Demographic, Clinical, and Sleep Characteristics of the Participants

Characteristic	Patients, *n* = 38		Control Subjects, *n* = 38
	Unmedicated	7-Day Medicated	
Age, Years, Mean ± SD	31.3 ± 10.2	–	31.6 ± 10.4
Gender Ratio, Female/Male, *n*	18/20	–	21/17
HAM-D, Mean ± SD	19.9 ± 3.8	15.2 ± 4.8	–
No. of Previous Depressive Episodes, Mean ± SD	1.76 ± 3.0	–	–
Sleep Stages, Minutes (%)			
N1	52.38 (12.4%)	62.53 (15.0%)	52.34 (12.4%)
N2	186.51 (44.1%)	201.03 (48.0%)	195.11 (46.2%)
N3	76.64 (18.4%)	70.74 (17.2%)	82.96 (19.9%)
REM	68.93 (16.3%)	49.54 (11.8%)^b^^,^^c^	69.34 (16.3%)
WASO	37.83 (8.8%)^a^	33.28 (8.0%)^b^	21.82 (5.2%)
Total Non-REM Sleep Time, Minutes (%)	263.16 (68.2%)	271.76 (70.6%)	278.07 (69.7%)
Total Sleep Time, Minutes, Mean ± SD	384.47 ± 40.0	383.83 ± 38.8	399.75 ± 39.4
Sleep Onset, Minutes, Mean ± SD	24.1 ± 30.8	20.9 ± 13.8	15.7 ± 9.6
N3 Onset, Minutes, Mean ± SD	24.91 ± 24.0	24.14 ± 19.8	18.21 ± 8.4
REM Onset, Minutes, Mean ± SD	98.61 ± 52.6	157.63 ± 79.6^b^^,^^c^	85.41 ± 34.8

Sleep stage percentages are given with respect to total sleep time. Non-REM sleep was defined as the combination of stages N2 and N3 without N1 sleep.

HAM-D, Hamilton Depression Rating Scale; REM, rapid eye movement; WASO, wakefulness after sleep onset.

a Significant difference between control subjects and unmedicated patients.

b Significant difference between control subjects and medicated patients.

c Significant difference between the unmedicated and medicated states of the patients.

Exclusion criteria included suicidality, shift working, transmeridian flights in the preceding 3 months, drug or alcohol dependence, professional piano skills, professional typewriting skills, sleep disorders, pregnancy, and a history of severe physical disorders. Subjects who received long-acting medication before the beginning of the experiment were excluded if the treatment was not stopped in time to ensure a complete washout (e.g., antipsychotics, fluoxetine). Because of technical failure in the EEG data of 2 medicated patients, all paired analyses were matched on the remaining full datasets (*n* = 38 per group).

To confirm the results, we replicated the analyses using 2 independently collected datasets of short- and long-term medicated patients with MDD (Supplementary Material 5). All studies were approved by the Ethics Committee of the University of Munich. All participants gave written informed consent.

### Questionnaires

Depression severity of patients was measured with the Hamilton Depression Rating Scale (HAM-D) at baseline (unmedicated) and 7 days after the commencement of antidepressant treatment (medicated). A higher score reflects higher depression severity. In Supplementary Material 4, we also report the Pittsburgh Sleep Quality Index, which was available in a subset of the patients.

### Polysomnography

All participants slept in the sleep laboratory, and all had an adaptation night before the examination night. For the EEG of the examination night, 118 Ag/AgCl electrodes were applied using an EasyCAP 128Ch-BrainCap. Polysomnography was recorded (sampling rate of 200 Hz), stored, and digitized following the 10-5 system (23) with a JE-209A amplifier (Neurofax Software; Nihon Kohden Europe GmbH, Rosbach, Germany) with a common-mode rejection ratio of ≥110 dB and with impedances below 10 kΩ, including EEG (filtered at 0.016-Hz high pass only, −6 dB/octave), electrooculography, and mental/submental electromyography with a ground electrode attached at the forehead. During the recording, the EEG was referenced to the average of the AFF5h and AFF1h, which were predefined by the hardware setup. For the offline analysis, the data were re-referenced to the average of all electrodes.

Polysomnography of the patients was recorded at 2 time points: when unmedicated and when medicated for 7 days. Sleep was scored by independent experts according to the American Academy of Sleep Medicine standards (24). We analyzed separately all sleep stages and the wakefulness after sleep onset (WASO). The epochs scored as the wake before the sleep onset and after morning awakening were excluded from the analysis, as they were not available for all participants. Epochs with electromyography and EEG artifacts and channels with more than 20% artifacts during non–rapid eye movement (non-REM) sleep were manually excluded by an experienced scorer before all automatic analyses. The rejection percentage is reported in Table S6.1.

In Supplementary Material 3, we also report morning resting-state EEG measured in a subset of participants to explore whether the observed effects are specific to sleep.

### Spectral Power

Total EEG power was differentiated into its aperiodic (fractal) and oscillatory components using the irregularly resampled auto-spectral analysis (25). A MATLAB 2021b (The MathWorks, Inc.) implementation of the algorithm was adapted from the FieldTrip website (http://www.fieldtriptoolbox.org/example/irasa). Specifically, we used the *ft_freqanalysis* function with the *cfg.method=‘irasa’* for each 30 seconds of sleep, corresponding to the conventionally defined sleep epochs. The function was called twice, with the *cfg.output=‘fractal’* and *cfg.output=‘original’* for the total power and its aperiodic component, respectively. The aperiodic component was transformed to log-log coordinates by standard least-squares regression, in which the slope of the line was calculated as the power-law exponent estimation.

Power was averaged over each sleep stage as defined by the hypnogram over 5 topographical areas: 1) frontal (Fz, F1, F2, F3, F4, F5, F6, F7, F8, F9, F10), 2) central (Cz, C1, C2, C3, C4, C5, C6), 3) parietal (Pz, P1, P2, P3, P4, P5, P6, P7, P8, P9, P10), 4) occipital (Oz, O1, O2), and 5) temporal (T7, T8).

The signal was filtered in the 0.2- to 48-Hz frequency band. In Supplementary Material 1, we also analyze low (2–20 Hz) and high (30–48 Hz) bands to control for a possible distortion of the linear fit by excluding low frequencies with strong oscillatory activity (15) and for the reliable discrimination between wakefulness and REM sleep, respectively (16).

In Supplementary Material 2, we report the analysis of the oscillatory component to explore whether the effect is specific to aperiodic activity.

### Statistical Analysis

To analyze aperiodic slopes, we used 5 analyses of covariance for each sleep stage separately with the 5-level brain area as the within-subjects factor and 2-level study group as the between-subjects factor to compare 1) unmedicated patients and control subjects and 2) the same patients when medicated for 7 days and control subjects. Even though we matched the participants’ ages individually, given that at the group level the age ranged from 19 to 54 years, we added to the analysis the age factor as a covariate. In view of between-group differences in the proportions of sleep stages (Table 1), when appropriate (namely, for WASO and REM), we added to the analyses of covariance the proportion of a given sleep stage in each study group as an additional covariate. We performed 5 additional analyses of variance for each sleep stage separately to compare unmedicated and 7-day medicated states of the patients using the state as the within-subjects factor.

The Benjamini-Hochberg adjustment was applied to control for multiple comparisons (5 tests reflecting the number of sleep stages) with a false discovery rate set at .05 and the α level set in the .01 to .05 range. For all analyses of variance/analyses of covariance, we applied Greenhouse-Geisser correction because Mauchly's test revealed that the sphericity assumption was violated (ε < 0.75, *p* < .05). The assumptions of normality and homogeneity of variance were tested using the Q-Q plot and Levene's homogeneity test, respectively.

Then, we performed post hoc analysis to compare each pair of groups for each area and sleep stage separately. We used the two-tailed Student unpaired *t* test to compare patients with control subjects and paired *t* test to compare the unmedicated and medicated states of the patients. Effect sizes were calculated with Cohen's *d*.

To study the effect of the antidepressant treatment on aperiodic activity, we stratified the patients by 1) antidepressant class and 2) REM-suppressive versus REM-nonsuppressive antidepressants as reported in Table 2. Then, we performed 25 nonparametric two-tailed Mann-Whitney *U* tests for each sleep stage and area separately, as after this stratification the samples were too small to perform analysis of variance. Benjamini-Hochberg adjustment for 25 tests (5 stages by 5 areas) was applied with the α level set in the .002 to .050 range.

**Table 2:** Demographic and Clinical Characteristics of the Subgroups of Patients by Medication Class

Medication Class	Sample Size, *n*	Age, Years, Mean ± SD	Female, *n*	No. of Previous Depressive Episodes, Mean ± SD	HAM-D Baseline, Mean ± SD	HAM-D 7 Days, Mean ± SD
SSRI (Citalopram, Escitalopram, Paroxetine, Sertraline)	13	29.9 ± 10.0	8	0.6 ± 0.8	19.7 ± 4.2	13.9 ± 4.6
TCA (Trimipramine, Amitriptyline, Amitriptylinoxide)	8	36.6 ± 11.9	4	2.1 ± 1.1	22.1 ± 3.4	16.6 ± 5.5
NDRI (Bupropion)	6	30.7 ± 10.5	3	0.7 ± 0.5	18.5 ± 3.5	17.8 ± 3.2
SNRI (Venlafaxine, Duloxetine)	6	31.7 ± 10.9	2	1.7 ± 0.8	18.3 ± 2.5	14.7 ± 5.5
NaSSA (Mirtazapine)	5	26.8 ± 6.1	3	2.6 ± 3.2	20.2 ± 4.8	13.8 ± 4.7
REM Suppressive (SSRI, SNRI, Amitriptylin, Amitriptylinoxide)	21	31.1 ± 10.3	11	1.1 ± 1.0	19.2 ± 3.6	14.1 ± 4.5
REM Nonsuppressive	17	31.6 ± 10.4	7	1.7 ± 2.0	20.7 ± 4.1	16.6 ± 4.9

The NaSSA subgroup was not analyzed separately due to a small sample size.

HAM-D, Hamilton Depression Rating Scale; NaSSA, noradrenergic and specific serotonergic antidepressant; NDRI, norepinephrine-dopamine reuptake inhibitor; REM, rapid eye movement; SNRI, serotonin and norepinephrine reuptake inhibitor; SSRI, selective serotonin reuptake inhibitor; TCA, tricyclic antidepressant.

The diagnostic accuracy of the frontal aperiodic slopes was defined using the area under the receiver operating characteristic curve. Pearson correlations were used to assess associations between the frontal aperiodic slopes on one side and 1) features of sleep architecture and 2) HAM-D scores of the participants at baseline and 7 days on the other side. SPSS software (version 25; IBM Corp.) was used for all statistical analyses.

## Results

The demographic, clinical, and sleep characteristics of the participants are reported in Table 1. Patients in both the unmedicated and medicated states showed increased WASO compared with control subjects. Medicated patients further showed decreased REM sleep proportion and prolonged REM sleep onset compared with the control subjects and their own unmedicated state.

### Aperiodic Slopes

**Figure 1:**
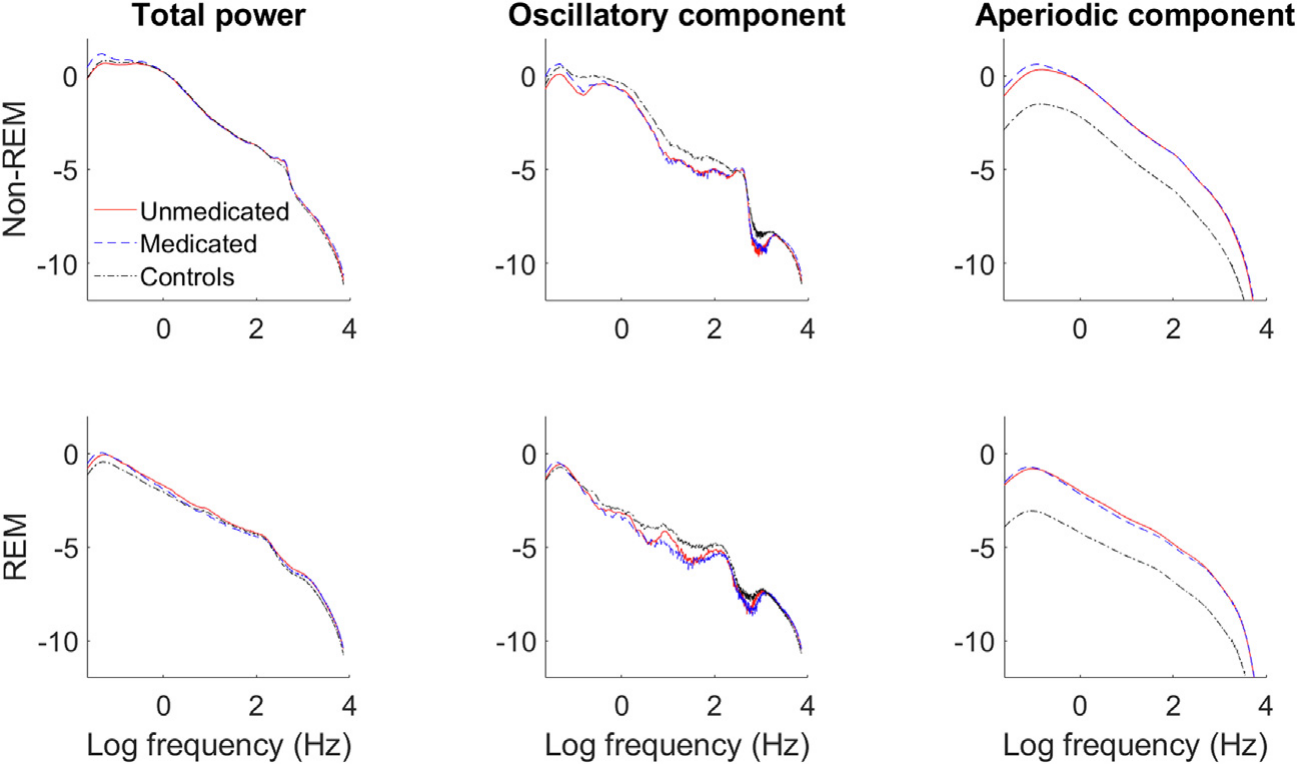
Electroencephalographic (EEG) power components. Total EEG spectral power (left) and its aperiodic (right) and oscillatory (middle) components averaged over frontal electrodes are plotted in the log-log space as a function of frequency for non–rapid eye movement (non-REM) (N2 + N3, top row) and REM (bottom row) sleep for each study group (different lines). Patients in both unmedicated (red lines) and medicated states (blue lines) show decreased oscillatory activity and steeper decay of the aperiodic component compared with control subjects (black lines). The total spectral power is comparable in all groups (coinciding lines of the left subgraphs).

**Figure 2:**
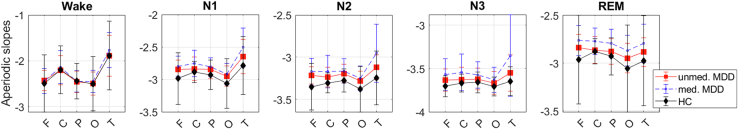
Aperiodic slopes. Slopes of the broadband (0.2–48 Hz) aperiodic power component over each sleep stage and area for each study group. Unmedicated (unmed.) patients (*n* = 38, red) show flatter (more positive values) slopes during N2 compared with healthy control subjects (HC, *n* = 38, black). Seven-day-medicated (med.) patients (*n* = 38, blue) show flatter slopes compared with their own unmedicated state (red) and control subjects (black) during all sleep stages—but not the wakefulness after sleep onset. C, central electrodes; F, frontal electrodes; MDD, major depressive disorder; O, occipital electrodes; P, parietal electrodes; REM, rapid eye movement; T, temporal electrodes.

Frontal total spectral power and its components for all sleep stages for all study groups are shown in Figure 1. The results are presented in Table 3 and Figure 2.

**Table 3:** Aperiodic Slopes

Stage	Unmedicated MDD Group, Mean					7-Day-Medicated MDD Group, Mean					HC Group, Mean				
	F	C	P	O	T	F	C	P	O	T	F	C	P	O	T
Wake	−2.43	−2.20	−2.46	−2.50	−1.89	−2.44	−2.14	−2.45	−2.45	−1.77	−2.49	−2.20	−2.44	−2.50	−1.89
N1	−2.84	−2.83	−2.84	−2.95	−2.65	−2.79	−2.75	−2.81	−2.91	−2.50	−2.98	−2.88	−2.92	−3.06	−2.78
N2	−3.22	−3.23	−3.19	−3.28	−3.12	−3.17	−3.17	−3.16	−3.25	−2.95	−3.35	−3.31	−3.28	−3.38	−3.25
N3	−3.63	−3.63	−3.62	−3.67	−3.55	−3.57	−3.55	−3.57	−3.63	−3.35	−3.70	−3.67	−3.66	−3.71	−3.64
REM	−2.84	−2.86	−2.88	−2.95	−2.88	−2.76	−2.77	−2.79	−2.87	−2.79	−2.96	−2.88	−2.93	−3.05	−2.97

	Unmedicated MDD-HC Comparison, Effect Size					Medicated MDD-HC Comparison, Effect Size					Unmedicated-Medicated MDD Comparison, Effect Size				
Stage	F	C	P	O	T	F	C	P	O	T	F	C	P	O	T
Wake	0.14	−0.01	−0.04	0.02	0.00	0.11	0.14	−0.03	0.12	0.20	−0.03	0.14	0.01	0.13	0.24
N1	0.46^a^	0.29	0.44^a^	0.40	0.37	0.62^a^	0.71^a^	0.64^a^	0.50^a^	0.74^a^	0.39^a^	0.48^a^	0.31	0.29	0.47^a^
N2	0.64^a^	0.62^a^	0.66^a^	0.46^a^	0.50^a^	0.79^a^	0.86^a^	0.81^a^	0.58^a^	0.89^a^	0.35^a^	0.42^a^	0.27	0.29	0.51^a^
N3	0.58^a^	0.31	0.31	0.34	0.50^a^	0.86^a^	0.73^a^	0.61^a^	0.59^a^	0.84^a^	0.37^a^	0.49^a^	0.40^a^	0.29^a^	0.45^a^
REM	0.37	0.10	0.31	0.31	0.26	0.57^a^	0.78^a^	0.78^a^	0.54^a^	0.50^a^	0.84^a^	1.18^a^	1.04^a^	1.15^a^	0.57^a^

	Unmedicated MDD-HC Comparison, ANCOVA						Medicated MDD-HC Comparison, ANCOVA						Unmedicated-Medicated MDD Comparison, ANOVA					
	Group		Area		Interaction		Group		Area		Interaction		Group		Area		Interaction	
Stage	*F(1,74)*	*p*	*F*(4,71)	*p*	*F*(1,4)	*p*	*F*(1,74)	*p*	*F*(4,71)	*p*	*F*(1,4)	*p*	*F*(1,74)	*p*	*F*(4,71)	*p*	*F*(1,4)	*p*
Wake^b^	0.2	.67	28.6	.00	0.3	.78	0.9	.34	25.7	.00	1.8	.14	1.0	.32	131.2	.00	2.5	.07
N1	3.4	.07	5.7	.01	1.3	.30	9.2	.00^a^	4.7	.01	3.8	.03	7.9	.01^a^	18.1	.00	6.1	.01
N2	7.2	.01^a^	3.8	.03	1.2	.30	14.4	.00^a^	3.5	.03	6.5	.00	6.6	.02^a^	54.9	.00	8.0	.00
N3	4.3	.04	1.4	.25	2.3	.10	14.9	.00^a^	0.5	.60	8.8	.00	7.9	.00^a^	18.1	.00	6.1	.01
REM^b^	1.8	.18	0.9	.50	1.5	.23	5.1	.03^a^	1.0	.40	1.8	.18	38.9	.00^a^	31.6	.00	20.6	.00

ANCOVA, analysis of covariance; ANOVA, analysis of variance; C, central electrodes; F, frontal electrodes; HC, healthy control; MDD, major depressive disorder; N, non–rapid eye movement stage; O, occipital electrodes; P, parietal electrodes; REM, rapid eye movement; T, temporal electrodes.

a Statistically significant *p* values after the correction for multiple comparisons; effect sizes were interpreted as small (0.2–0.5), medium (0.5–0.8), and large (0.8–1.2).

b ANCOVAs adjusted for the proportion of the corresponding sleep stage.

Unmedicated patients showed flatter slopes compared with control subjects during N2 sleep (*p* = .009) in all areas and during N3 sleep (*p =* .04) in the frontal and temporal areas with medium effect sizes.

Medicated patients showed flatter slopes compared with control subjects during N1, N2, N3, and REM sleep in all areas with medium effect sizes. These findings were replicated in 2 independent datasets (Supplementary Material 5).

Patients in the medicated state showed flatter slopes compared with their own unmedicated state with a large effect size during REM sleep and with a small effect size during stage N3. Likewise, the medicated state showed flatter slopes during stages N1 and N2 in the frontal, central, and temporal areas with small effect sizes compared with the unmedicated state (Figure 2 and Table 3).

Aperiodic slopes did not correlate with depression severity (HAM-D scores) at the baseline and 7-day assessments.

### Receiver Operating Characteristic Analysis

Frontal slopes measured during N1, N2, N3, and REM sleep discriminated both between the unmedicated state of the patients and control subjects (area under the receiver operating characteristic curve, 0.66–0.74; all *p* values < .02) and between the medicated state of the patients and control subjects (area under the receiver operating characteristic curve, 0.74–0.76; all *p* values < .001).

### Medication Effect

**Figure 3:**
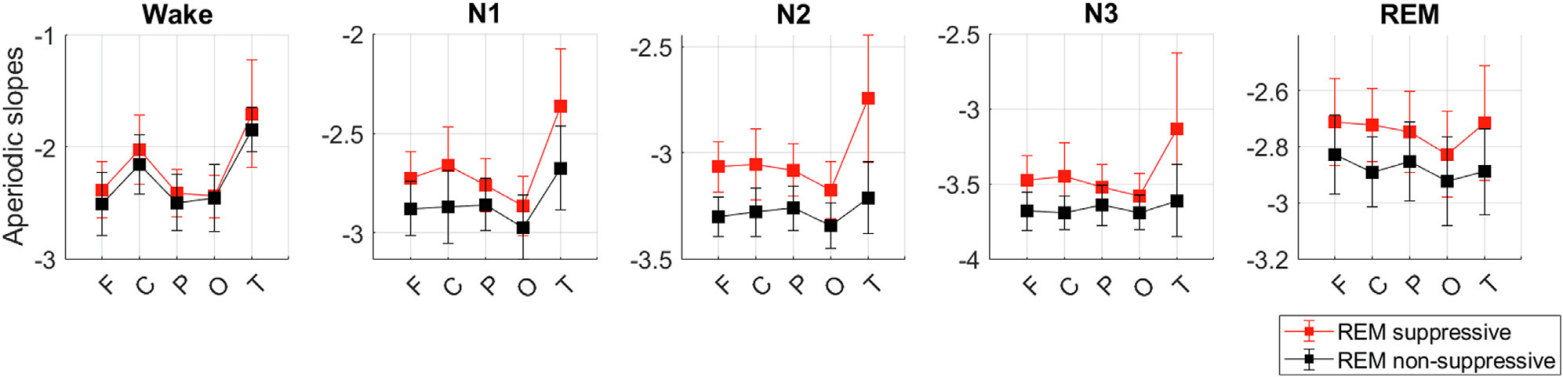
Effect of rapid eye movement (REM)–suppressive medications. Slopes of the aperiodic power component in the 0.2- to 48-Hz frequency band were averaged over each sleep stage over each area. Patients who took REM-suppressive antidepressants for 7 days (*n =* 21, red) showed flatter slopes (higher values) than patients who took REM-nonsuppressive antidepressants for 7 days (*n =* 17, black) during all sleep stages but not the wakefulness after sleep onset. C, central electrodes; F, frontal electrodes; N, non–rapid eye movement stage; O, occipital electrodes; P, parietal electrodes; T, temporal electrodes.

The demographic and clinical characteristics of the subgroups of patients stratified by medication class are reported in Table 2. The results are presented in Figure 3 and Supplementary Material 5 (Table S5.5).

The patients who took REM-suppressive antidepressants showed flatter slopes than patients who took REM-nonsuppressive antidepressants during N1 (*F(1, 36)* *=* 13.9, *p =* .001), N2 (*F(1, 36)* *=* 42.8, *p <* .001), N3 (*F(1, 36)* *=* 21.9, *p <* .001), and REM (*F(1, 36)* *=* 10.4, *p =* .003) sleep, with large effect sizes (Cohen’s *d* values = 0.6–2.2).

The patients who took REM-suppressive serotonin and norepinephrine reuptake inhibitors (SNRIs) showed flatter slopes during N2, N3, and REM sleep with large to huge effect sizes than the patients who took non-SNRIs (*p* values < .001–.026, Cohen’s *d* values = 0.9–2.8). These patients also showed flatter slopes than the patients who took non-SNRIs and REM-suppressive antidepressants (*p* values = .005–.019, Cohen’s *d* values = 1.5–1.7), such as selective serotonin reuptake inhibitors (SSRIs) (*p* values = .009–.028, Cohen’s *d* values = 1.3–1.8) during REM sleep with very large effect sizes. However, these findings did not pass the correction for multiple comparisons. Aperiodic activity was comparable among patients who took SSRIs, tricyclic antidepressants, or norepinephrine and dopamine reuptake inhibitors compared with their pooled control subjects.

### Aperiodic Slopes and Sleep Architecture

Correlations between aperiodic slopes and sleep architecture are reported in Table 4. In unmedicated patients, flatter aperiodic slopes during stage N1 were associated with the delayed onset of stage N3. In medicated patients, flatter aperiodic slopes during non-REM sleep were linked to the higher proportion of N1, lower proportion of REM, delayed onset of N3 and REM, and shorter total sleep time. In control subjects, flatter aperiodic slopes during N3 sleep were associated with the higher proportion of WASO and lower proportion of REM sleep. Some of these findings were replicated in an independent dataset (Supplementary Material 5, Table S5.2).

**Table 4:** Correlations Between Aperiodic Slopes and Sleep Architecture

Pair for Correlation	Patients		Control Subjects
	Unmedicated	Medicated	
N1 Slope–N1%	n.s.	0.347^a^	n.s.
N1 Slope–SWS Onset	0.335^a^	0.351^a^	n.s.
N2 Slope–N1%	n.s.	0.479^b^	n.s.
N2 Slope–REM%	n.s.	−0.548^b^	n.s.
N2 Slope–REM Onset	n.s.	0.391^a^	n.s.
N2 Slope–TST	n.s.	−0.328^a^	n.s.
N3 Slope–WASO	n.s.	n.s.	0.328^a^
N3 Slope–N1%	n.s.	0.455^b^	n.s.
N3 Slope–REM%	n.s.	−0.357^a^	−0.591^b^

Sleep stage percentages were calculated with respect to TST, REM, non-REM, and WASO. Pearson correlation coefficients (*r*) between aperiodic slopes measured during a particular sleep stage and features of sleep architecture are presented for each group separately. Only the *r*s associated with the statistically significant *p* values are presented, i.e., the rest of the possible combinations between aperiodic slopes and sleep architecture features were statistically nonsignificant.

N, non–rapid eye movement stage; n.s., not significant; REM, rapid eye movement; SWS, slow wave sleep; TST, total sleep time; WASO, wakefulness after sleep onset.

a .05 > *p* > .01.

b *p* < .01.

The alterations reported in the Results were specific to sleep and were not observed during the morning resting state (Supplementary Material 3).

## Discussion

To the best of our knowledge, this is the first study to examine sleep-related aperiodic activity in MDD and its relationships with sleep architecture, depression severity, and responsivity to antidepressant treatment. We found that unmedicated patients showed flatter aperiodic slopes during non-REM sleep than control subjects. Patients in the medicated state showed flatter aperiodic slopes compared with their own unmedicated state and healthy control subjects during both non-REM and REM sleep. In medicated patients, flatter aperiodic slopes during non-REM sleep were linked to the lower proportion and delayed onset of REM sleep. We replicated several of our findings in 2 independently collected datasets of medicated patients. Below, we aim at an interpretation of these findings.

The functional significance of aperiodic dynamics is still a mystery, with several interpretations suggested so far. For example, aperiodic activity can manifest in the overall firing rate of cortical neurons (9,15,26) as measured by local field potentials or EEG. When many neurons fire relatively simultaneously, the power spectrum will decay faster, being relatively stronger in low frequencies and relatively weaker in the higher ones. Mathematically, this will be expressed by a more negative (steeper) slope, which in turn reflects a higher power-law exponent. A steeper slope can signify redundancy (11), excessive or insufficient propagation of the signal (27), or increased dendritic filtering (26). When neurons fire relatively asynchronously, the spectral power is shifted toward higher frequencies and its slope is flatter, reflecting reduced temporal autocorrelations (8), a high entropy rate of cortical systems (28), or a noisier neural background (29, 30, 31). Following this, flatter aperiodic slopes observed here in unmedicated and medicated patients with MDD may reflect noisier neural background activity (31), which in turn can adversely affect sleep and its restorative function.

Adding to this, pharmacological, physiological, and computational studies linked aperiodic activity to the balance between excitatory and inhibitory currents in the brain (15,16). Specifically, Gao *et al.* (15) showed that the aperiodic slope in the 30- to 50-Hz range reliably tracked the induction and the recovery from propofol-induced anesthesia in rats and macaques. Similarly, in humans, inhibition was boosted by the propofol administration, and the slope became steeper when inhibition increased (16). Subsequent studies interpreted the 1/f exponent as an indicator of E/I balance also for other frequency bands, e.g., 3 to 55 Hz (10), 0.5 to 35 Hz (31), 1 to 40 Hz, 1 to 20 Hz, and 20 to 40 Hz (32). In Supplementary Material 1, we analyzed aperiodic activity in the 2- to 20-Hz and 30- to 48-Hz bands and confirmed the broadband (0.2–48 Hz) analysis reported in the main text with the exception of the high-band activity, which was comparable in unmedicated patients and control subjects.

The right balance between neural E/I is crucial for optimal signal formation and transmission, synaptic plasticity, neuronal growth, and pruning and, thus, enables flexible behavior and cognition (17). Correspondingly, any perturbations in the E/I balance may lead to brain disease (17). For example, schizophrenia has been associated with a low E/I ratio caused by hypoactive receptors for the excitatory neurotransmitter glutamate (17) and steeper aperiodic slopes compared with control subjects during rest (33). Analogously, depression might be associated with E/I perturbations due to its cholinergic-monoaminergic (18,19), glutamatergic (20), and/or GABAergic imbalance.

Thus, it has been suggested that GABAergic deficit may play a central role in the etiology of MDD, especially in melancholic (21) and treatment-resistant (22) types of depression, while targeting the E/I imbalance in depression via enhancing the GABAergic system with antidepressant therapies may contribute to a greater remission rate and reduce the risk of relapse (34). At the cellular level, changes in GABAergic interneurons affect the regulation of excitatory signals from and onto pyramidal neurons (35), the primary contributors to the EEG signal. Following this literature, flatter aperiodic slopes observed here during sleep in unmedicated and medicated patients may reflect a shift in the E/I ratio in favor of excitation due to cellular alterations of the GABAergic, glutamatergic, and cholinergic-monoaminergic systems. Nevertheless, it should be stressed that thus far there is no evidence that altered aperiodic dynamics can be used as readouts of cholinergic, monoaminergic, glutamatergic, and GABAergic imbalance. Moreover, whereas some authors have suggested that the aperiodic slope is an indicator of E/I balance (10,15,16), others have stated that currently the relationship between aperiodic slopes and E/I balance remains a hypothesis to be further validated (7). In addition, when aiming to link aperiodic slopes and E/I balance, one should keep in mind that aperiodic 1/f-like processes are very ubiquitous in nature and are not limited to neural activity (7,10,11).

Of special interest was the effect of antidepressants: we found that during all sleep stages, medicated patients showed flatter slopes compared with control subjects. We replicated this association using 2 independently collected datasets of short- and long-term-medicated patients (Supplementary Material 5). In addition, we found that the medicated state showed flatter slopes compared with the patients’ own unmedicated state. In line with our findings, a recent study in healthy women reported that 1 week of intake of the SSRI escitalopram induced a flattening of aperiodic slopes during rest in favor of excitation (36).

Furthermore, we found that in 7-day-medicated patients, flatter aperiodic slopes during non-REM sleep correlated with such alterations in sleep architecture as a higher proportion of N1, a lower proportion of REM, delayed onset of N3 and REM sleep, and shorter total sleep time. In the literature, these alterations in sleep architecture are often interpreted as impaired sleep (37). Specifically, patients with MDD have been reported to show prolonged sleep latency, increased WASO, early-morning awakening, reduced slow-wave sleep, shortened latency, and increased amount of REM sleep (38). Here, unmedicated patients with MDD have shown increased WASO, while other sleep architecture features were comparable to those measured in control subjects. The same patients in the medicated state showed increased WASO, decreased REM sleep proportion, and prolonged REM sleep onset.

Delayed onset and reduced amount of REM sleep are well-known aftereffects of almost all antidepressants (38). Notably, we replicated the association between flatter aperiodic slopes during non-REM sleep and a decreased proportion of REM sleep and delayed REM sleep onset using an independent dataset of 7-day-medicated patients (Supplementary Material 5, Table S5.2). This link is in line with the previous proposition that some antidepressants (for example, such as the SNRI venlafaxine) may impair sleep due to their activating effects (39). Furthermore, the observed association suggests that aperiodic slope flattening seen in medicated patients with MDD is a potential readout of altered sleep architecture and impaired sleep known in this disorder. Nevertheless, further studies are needed to test this possibility.

Whereas our findings bring new insights about the association between aperiodic activity, sleep architecture, and antidepressants, they do not advance the current understanding of the treatment response (or lack thereof), as changes in aperiodic activity did not correlate with clinical improvement (as assessed by the HAM-D). It is possible that other depression scales (that were not available in this study) would be more sensitive in detecting the hypothesized association between depression severity and aperiodic activity. Given that a deeper understanding of the antidepressants' effects on sleep is crucial for successful treatment, future large-scale longitudinal research is required to reveal whether aperiodic activity can serve as a marker for predicting individual cortical responsivity to different antidepressants.

Besides their clinical importance, our findings are also essential from the methodological point of view, as they confirm the importance of a recent recommendation to differentiate the total spectral power to its components in order to avoid misrepresentation and misinterpretation of the data (8,9). Namely, we observed comparable total (i.e., nondifferentiated to its components) spectral power (analyzed in 6) but different oscillatory and aperiodic components in unmedicated patients with MDD and control subjects (Figure 1).

Finally, here, aperiodic alterations were observed during sleep but not during WASO, suggesting their specificity to sleep. Nevertheless, it should be kept in mind that the wake EEG is more variable and prone to artifacts than the sleep EEG; therefore, the performed analysis might have not enough statistical power to detect between-group differences. Interestingly, in unmedicated patients, the morning resting-state EEG showed steeper low-band and flatter high-band slopes compared with control subjects, while broadband slopes were comparable in both groups. This preliminary finding, however, requires further validation due to the small size of the tested sample (16 patients vs. 16 control subjects) (Supplementary Material 3).

This study is not without limitations. First, one should keep in mind that the diagnosis of depression is subjective, and subtypes of depression likely exist even though they have not been systematically distinguished. Likewise, the stratification of the patients by antidepressant classes performed here was not clean enough, as the patients used different antidepressants. Second, this research is correlational and precludes causal relations between the neurobiology of MDD and aperiodic activity.

In conclusion, our findings suggest that flatter aperiodic slopes represent a new disease-relevant feature of sleep in MDD, which may reflect unstable, noisy neural activity due to a shift of the E/I ratio in favor of excitation. In the future, these findings may lead to the development of a biomarker for personalized disease monitoring and therapy.

## Supplementary Material

Supplemental Information

Key Resources Table
