# Supplementary material for: Increased Aperiodic Neural Activity During Sleep in Major Depressive Disorder
Source: Biol Psychiatry Glob Open Sci. 2022 Oct 25;3(4):1021–9. doi: 10.1016/j.bpsgos.2022.10.001 (PMC10593867; doi:10.1016/j.bpsgos.2022.10.001)
Supplement: Supplemental Information [file mmc1.pdf]

# SUPPLEMENTAL INFORMATION

# Increased Aperiodic Neural Activity During Sleep in Major Depressive Disorder

Rosenblum *et al.*

# Supplementary Material 1

## Low and high band aperiodic power

In the Main Text, we analyzed the broadband (0.2-48Hz) signal. Here, we further report low (2-20Hz) and high (30-48Hz) frequency bands analyses. The low-band analysis was added to this work to control for a possible distortion of the linear fit of the aperiodic component by excluding low frequencies with residual (failed to be excluded by the Irregularly Resampled Auto-Spectral Analysis algorithm) strong oscillatory activity as well as the so-called "knees", specific bends seen on the power spectrum in log-log space around 1-2Hz and 20Hz (2). We added the high-band analysis inspired by the literature reporting that this band facilitates the reliable discrimination between wakefulness and REM sleep (3). The analysis was limited to 48 Hz due to line noise in recordings (50Hz in Europe) and broadband muscle artifacts (3).

**Results**

*Low-band slopes*

The results are presented in Table S1.1 and Fig.S1.1. Unmedicated patients showed flatter slopes compared to controls during N2 and N3 sleep in all areas with medium effect sizes. Medicated patients showed flatter slopes during N2, N3, and REM sleep in all areas with medium effect sizes compared to controls. During N1 sleep, medicated patients showed flatter slopes in the central, parietal, and temporal areas with medium effect sizes compared to controls.

Patients showed flatter slopes when medicated than when unmedicated during N3 and REM sleep in all areas with medium effect sizes, and during the N1 and N2 stages – in the central and temporal areas with medium effect sizes. Slopes during the wakefulness after sleep onset were comparable in all groups.

*High-band slopes*

The results are presented in Table S1.1 and Fig.S1.1. Unmedicated patients and controls showed comparable high-band slopes. Medicated patients showed flatter slopes during N1 and N2 in the frontal, central, and occipital areas, and during N3 sleep in the frontal, central, and temporal areas with medium effect sizes compared to controls. Slopes during the wakefulness after sleep onset and REM sleep were similar to those measured in the control group. Patients showed flatter slopes when medicated than when unmedicated during all stages in all areas with medium effect sizes.

Notably, in the control group, the steepest high-band slopes were observed during the N2 sleep stage. This is contradictory to Lendner et. al (3) who report that the steepest slopes are observed during REM sleep, the findings which have been recently replicated in ~10,000 healthy individuals (4). Our result might reflect a statistical error of a random sample.

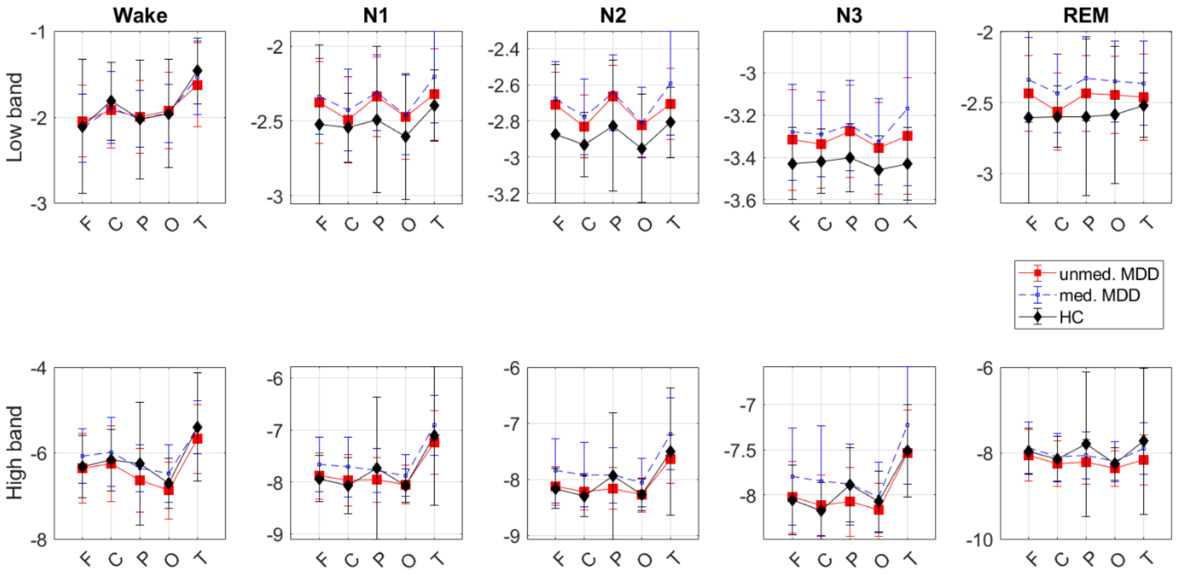


**Figure S1.1. Low and high-band aperiodic slopes.** Slopes of the aperiodic power component in the low (2-20Hz, **top**) and high (30-48Hz, **bottom**) frequency bands over each area of interest for each sleep stage and study group separately. **Top:** Unmedicated patients (red) show flatter (more positive values) low slopes during N2 and N3 stages compared to controls (black) in all areas. 7-day medicated patients (blue) show flatter low-band slopes compared to the own unmedicated state (red) and controls (black) during all sleep stages in all areas. **Bottom:** 7-day medicated patients (blue) show flatter high-band slopes compared to the own unmedicated state (red) during all sleep stages and compared to controls (black) during non-REM sleep. MDD – 38 major depressive disorder patients, unmed. – unmedicated, med. - 7-day medicated, HC – 38 healthy controls, F – frontal, C – central, P – parietal, O – occipital, T – temporal electrodes.

**Table S1.1: Low and high-band aperiodic slopes**

| Mean | Groups | Unmedicated MDD | | | | | 7-day medicated MDD | | | | | HC | | | | |
|---|---|---|---|---|---|---|---|---|---|---|---|---|---|---|---|---|
| Low band | Area/ Stage | F | C | P | O | T | F | C | P | O | T | F | C | P | O | T |
| | Wake | -2.05 | -1.91 | -1.99 | -1.92 | -1.62 | -2.13 | -1.89 | -2.02 | -1.96 | -1.54 | -2.11 | -1.81 | -2.03 | -1.96 | -1.46 |
| | N1 | -2.38 | -2.49 | -2.34 | -2.47 | -2.32 | -2.34 | -2.43 | -2.31 | -2.46 | -2.20 | -2.52 | -2.54 | -2.49 | -2.60 | -2.40 |
| | N2 | -2.71 | -2.83 | -2.66 | -2.83 | -2.71 | -2.68 | -2.78 | -2.64 | -2.81 | -2.59 | -2.87 | -2.93 | -2.83 | -2.95 | -2.81 |
| | N3 | -3.32 | -3.34 | -3.28 | -3.36 | -3.30 | -3.28 | -3.29 | -3.25 | -3.33 | -3.17 | -3.43 | -3.42 | -3.40 | -3.46 | -3.43 |
| | REM | -2.43 | -2.56 | -2.44 | -2.45 | -2.46 | -2.34 | -2.43 | -2.33 | -2.35 | -2.36 | -2.60 | -2.60 | -2.60 | -2.59 | -2.52 |
| High band | Area/ Stage | F | C | P | O | T | F | C | P | O | T | F | C | P | O | T |
| | Wake | -6.35 | -6.25 | -6.63 | -6.87 | -5.67 | -6.07 | -5.98 | -6.36 | -6.48 | -5.40 | -6.32 | -6.17 | -6.24 | -6.71 | -5.39 |
| | N1 | -7.89 | -7.97 | -7.96 | -8.05 | -7.24 | -7.66 | -7.71 | -7.79 | -7.88 | -6.91 | -7.93 | -8.07 | -7.74 | -8.07 | -7.12 |
| | N2 | -8.12 | -8.22 | -8.16 | -8.28 | -7.64 | -7.85 | -7.92 | -7.93 | -8.06 | -7.19 | -8.16 | -8.30 | -7.94 | -8.28 | -7.50 |
| | N3 | -8.03 | -8.12 | -8.08 | -8.17 | -7.54 | -7.80 | -7.85 | -7.88 | -8.03 | -7.23 | -8.06 | -8.18 | -7.89 | -8.08 | -7.51 |
| | REM | -8.05 | -8.24 | -8.20 | -8.36 | -8.15 | -7.88 | -8.09 | -8.05 | -8.20 | -7.89 | -7.96 | -8.15 | -7.80 | -8.26 | -7.73 |
| **Effect size** | **Groups** | **Unmedicated MDD-HC** | | | | | **Medicated MDD-HC** | | | | | **Unmedicated-medicated MDD** | | | | |
| Low band | Area/ Stage | F | C | P | O | T | F | C | P | O | T | F | C | P | O | T |
| | Wake | 0.10 | -0.22 | 0.06 | 0.06 | -0.37 | -0.03 | -0.17 | 0.01 | 0.00 | -0.20 | -0.20 | 0.05 | -0.08 | -0.07 | 0.15 |
| | N1 | 0.35 | 0.20 | 0.39 | 0.36 | 0.27 | 0.45 | **0.47** | **0.47** | 0.41 | **0.69** | 0.29 | **0.48** | 0.24 | 0.10 | **0.53** |
| | N2 | **0.54** | **0.57** | **0.57** | **0.52** | **0.52** | **0.63** | **0.79** | **0.62** | **0.56** | **0.88** | 0.31 | **0.51** | 0.18 | 0.17 | **0.51** |
| | N3 | **0.55** | **0.45** | **0.66** | **0.53** | **0.57** | **0.74** | **0.73** | **0.81** | **0.71** | **0.92** | **0.28** | **0.47** | **0.25** | **0.26** | **0.40** |
| | REM | 0.36 | 0.14 | 0.39 | 0.36 | 0.21 | **0.55** | **0.66** | **0.61** | **0.59** | **0.59** | **0.71** | **0.93** | **0.73** | **0.83** | **0.67** |
| High band | Area/ Stage | F | C | P | O | T | F | C | P | O | T | F | C | P | O | T |
| | Wake | -0.04 | -0.10 | -0.34 | -0.26 | -0.26 | 0.36 | 0.24 | -0.10 | 0.36 | 0.00 | **0.34** | 0.28 | **0.36** | **0.49** | **0.36** |
| | N1 | 0.11 | 0.20 | -0.22 | 0.05 | -0.12 | **0.57** | **0.65** | -0.05 | **0.52** | 0.20 | **0.49** | **0.42** | **0.45** | **0.48** | **0.47** |

| | | | | | | | | | | | | | | | | |
|---|---|---|---|---|---|---|---|---|---|---|---|---|---|---|---|---|
| | N2 | 0.12 | 0.23 | -0.26 | 0.00 | -0.15 | **0.66** | **0.78** | 0.02 | **0.59** | 0.34 | **0.57** | **0.56** | **0.61** | **0.63** | **0.69** |
| | N3 | 0.08 | 0.17 | -0.47 | -0.29 | -0.06 | **0.56** | **0.68** | 0.02 | 0.15 | 0.48 | **0.44** | **0.51** | **0.49** | **0.40** | **0.53** |
| | REM | -0.16 | -0.18 | -0.32 | -0.27 | -0.34 | 0.15 | 0.10 | -0.20 | 0.13 | -0.13 | **0.47** | **0.41** | **0.48** | **0.55** | **0.55** |

***Bold** font indicates statistically significant p-values after the correction for multiple comparisons, gray font indicates non-significant values, effect sizes are interpreted as small (0.2–0.5), medium (0.5–0.8), and large (0.8–1.2), MDD – major depressive disorder, HC – healthy controls, F – frontal, C – central, P – parietal, O – occipital, T – temporal electrodes, REM – rapid eye movement sleep, N – non–rapid eye movement sleep.*

# Supplementary Material 2

## Oscillatory analysis

Here, we explore oscillatory dynamics (Fig.1, middle) to assess whether the observed in the Main Text changes were specific to aperiodic activity.

**Methods**

Total EEG power averaged over all frontal electrodes was differentiated into its aperiodic (fractal) and oscillatory components using the Irregularly Resampled Auto-Spectral Analysis as described in the Methods of the Main Text. The oscillatory component was calculated by subtracting the aperiodic component from the total power. Then, the power was averaged over slow-wave activity (SWA, 0.2-4Hz), theta (4-8Hz), alpha (8-12Hz), and beta (15-30Hz) frequency bands.

We compared each pair of study groups for each sleep stage separately. We used the two-tailed Student's unpaired t-test to compare patients to controls and paired t-test to compare the unmedicated and medicated states of the patients. Effect sizes were calculated with Cohen's d. Due to the exploratory nature of this analysis and a high number of comparisons the α-level was set at 0.01.

Correlations between oscillations and HAM-D scores of the participants at baseline and 7-day were assessed with Pearson's correlation coefficients within each group separately.

## Results

The oscillatory component in different frequency bands for each sleep stage for each study group is presented in Table S2.1. Unmedicated patients showed decreased SWA and theta oscillations during N1, decreased SWA, theta, and beta oscillations during N2 and N3, and decreased theta oscillations during REM sleep compared to the controls.

Medicated patients showed decreased theta and beta oscillations during N2 and N3, and decreased theta oscillations during REM sleep compared to the controls.

Medicated and unmedicated states showed comparable oscillations (all p-values > 0.01).

In unmedicated patients, out of all parameters, only alpha-band oscillations during N2 positively correlated with depression severity (higher HAM-D scores, r=0.456, p=0.004). The rest of the oscillations at baseline or 7-day did not correlate with depression severity (HAM-D scores).

**Table S2.1. Oscillations**

| Sleep stage | Frequency band | Unmedicated | Medicated | Controls | Unmed-HC | | Med-HC | |
|---|---|---|---|---|---|---|---|---|
| | | | | | d | p | d | p |
| N1 | **SWA** | 0.052 | 0.076 | 0.111 | **-0.745** | **0.002** | -0.303 | 0.191 |
| | **Theta** | 0.007 | 0.007 | 0.013 | **-0.623** | **0.008** | -0.570 | 0.015 |
| | **Alpha** | 0.008 | 0.008 | 0.010 | -0.234 | 0.311 | -0.234 | 0.312 |
| | **Beta** | 0.001 | 0.001 | 0.001 | -0.290 | 0.210 | -0.363 | 0.118 |
| N2 | **SWA** | 0.102 | 0.147 | 0.212 | **-0.704** | **0.003** | -0.345 | 0.137 |
| | **Theta** | 0.007 | 0.008 | 0.016 | **-0.902** | **0.000** | **-0.821** | **0.001** |
| | **Alpha** | 0.007 | 0.007 | 0.010 | -0.442 | 0.058 | -0.378 | 0.104 |
| | **Beta** | 0.000 | 0.000 | 0.001 | **-0.676** | **0.004** | **-0.787** | **0.001** |
| N3 | **SWA** | 0.509 | 0.537 | 0.828 | -0.561 | 0.017 | -0.451 | 0.055 |
| | **Theta** | 0.012 | 0.011 | 0.027 | **-0.944** | **0.000** | **-0.985** | **0.000** |

| | | | | | | | | |
|---|---|---|---|---|---|---|---|---|
| | **Alpha** | 0.010 | 0.011 | 0.013 | -0.288 | 0.214 | -0.216 | 0.351 |
| | **Beta** | 0.000 | 0.000 | 0.000 | **-0.870** | **0.000** | **-0.988** | **0.000** |
| **REM** | **SWA** | 0.066 | 0.049 | 0.064 | 0.023 | 0.921 | -0.191 | 0.408 |
| | **Theta** | 0.006 | 0.005 | 0.012 | **-0.780** | **0.001** | **-0.991** | **0.000** |
| | **Alpha** | 0.005 | 0.005 | 0.008 | -0.457 | 0.050 | -0.541 | 0.021 |
| | **Beta** | 0.001 | 0.001 | 0.001 | -0.057 | 0.805 | -0.150 | 0.516 |

***Bold** font indicates p-values, effect sizes are interpreted as small (0.2–0.5), medium (0.5–0.8), and large (0.8–1.2). HC – healthy controls, unmed – unmedicated, med – medicated, REM – rapid eye movement sleep, N – non–rapid eye movement sleep, d – effect size calculated using Cohen's d*

# Supplementary Material 3

## Aperiodic activity during resting state

Here, we explore resting-state EEG to evaluate whether the reported in the Main text effects are specific to sleep or can be observable during wake as well.

Resting-state EEG was available in a subset of 16 unmedicated patients and 16 age-matched controls. The signal was recorded in the morning (~ 8 a.m.) following the patient's sleep in the lab. The participants were asked to close their eyes and sit quietly for 15 minutes, staying relaxed in a state of mind-wandering (i.e., no goal-oriented mental activity). Data was filtered using a band-pass finite impulse response filter 0.2-48Hz and divided into 1s epochs. Epochs with voltage > 50μV from the mean across all epochs were rejected from further analysis to reduce noise. The aperiodic component was calculated and analyzed as described in the Methods of the Main Text.

We found that at rest, unmedicated patients and controls showed comparable broadband aperiodic slopes. Low-band aperiodic slopes were steeper (more negative values) in the frontal (d=-0.8), central (d=-0.8), and temporal (d=-0.7) electrodes with medium to large effect sizes compared to controls. High-band aperiodic slopes were flatter (more negative values) in the central (d=0.9) and temporal (d=1.0) electrodes with large effect sizes compared to controls (Fig. S3.1).

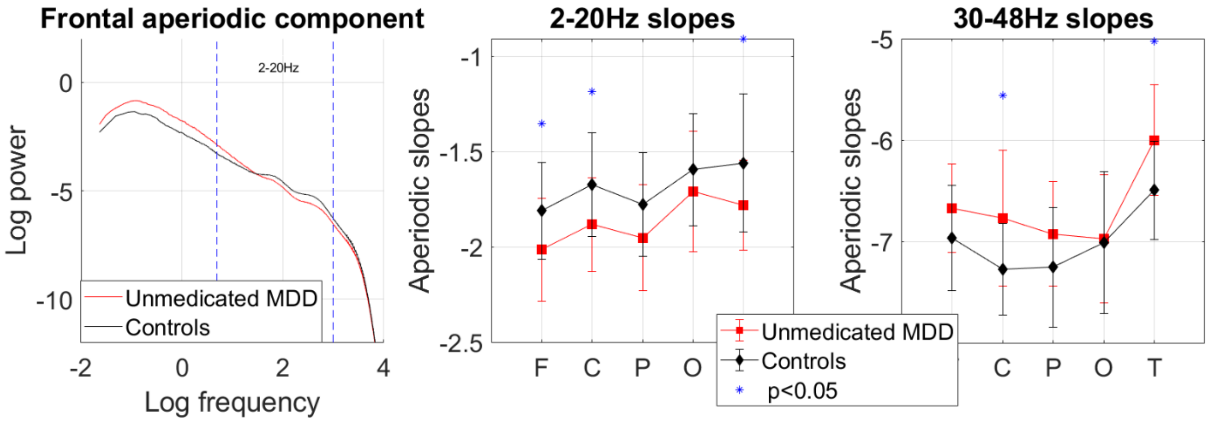


**Figure S3.1. Resting state aperiodic power component. Left:** aperiodic power component over the frontal electrodes. **Middle:** slopes of the aperiodic power component in the low (2-20Hz) band for each area of interest separately. **Right:** high (30-48Hz) band aperiodic slopes for each area of interest separately. The morning resting state showed comparable broadband slopes, steeper low-band slopes in the frontal, central and temporal electrodes, and flatter high-band slopes in the central and temporal areas in unmedicated patients (red) compared to controls (black). MDD – major depressive disorder patients, F – frontal, C – central, P – parietal, O – occipital, T – temporal electrodes, asterisks mark statistically significant p-values.

# Supplementary Material 4

## Aperiodic activity and sleep quality

Based on the literature reporting steeper slopes during deeper sleep stages (3, 5), we hypothesized that steeper slopes might reflect better sleep quality. We assessed correlations between aperiodic slopes and Pittsburgh Sleep Quality Index (PSQI). PSQI assesses sleep quality and disturbances over a one-month time interval and, therefore, is not a questionnaire of choice to correlate with a highly dynamic aperiodic activity. Unfortunately, other sleep questionnaires were not available in this study due to its retrospective nature.

A subset of 32 unmedicated patients and 37 controls filled in the PSQI, a self-rated questionnaire where a higher score reflects a worse subjective sleep quality. Associations between the aperiodic slopes and PSQI scores were assessed with Pearson's correlation coefficients in each group separately.

In both unmedicated patients and controls, sleep quality (the PSQI scores) did not correlate with aperiodic slopes.

# Supplementary Material 5

## Replication studies

To confirm the results reported in the Main study, here, we replicated the analysis reported in the Main Text using two independently collected datasets of short and long-term medicated patients with major depressive disorder (MDD) and healthy controls.

These datasets are described in our previous report, where Replication Dataset 1 is referred to as "Dataset C" (thirty MDD patients at 7-day and 28-day of medication treatment vs 28 healthy controls) and Replication Dataset 2 is referred to as "Dataset A" (forty long-termed medicated MDD patients vs forty healthy controls) (6).

Similar to the main study, here, we calculated the aperiodic slopes and compared them between the groups. In addition, we stratified the 7-day medicated patients from the Replication Dataset 1 into two subgroups by 1) the medication class: SNRIs (n = 12) and TCAs (n = 10); 2) REM-suppressive (n = 21) vs non-suppressive (n = 9) antidepressant type (Table S5.3). In Replication Dataset 1, the number of patients who took SSRIs (n = 2), NDRIs (n = 3), or NaSSA (n = 2) was too small, therefore, these subgroups were not analyzed separately. The effect of medication was not analyzed for Replication Dataset 2 since all patients there were long-term medicated and most of them took a combination of different drugs.

**Results**

The demographic, clinical, and sleep characteristics of the participants are reported in Table S5.1. Both datasets showed a similar distribution of age, depression severity (moderate to strong), and the number of previous depressive episodes (several, typically not the first).

**Table S5.1: Demographic, clinical, and sleep characteristics of the participants** (mean ± std)

| | **Replication Dataset 1** | | | **Replication Dataset 2** | |
|---|---|---|---|---|---|
| | **Controls** | **7-day medicated patients** | **28-day medicated patients** | **Controls** | **Medicated patients** |
| **No. of participants** | 28 | 30 | --- | 40 | 40 |
| **Age, years** | 45.5 ± 16.5 | 45.6 ± 16.8 | --- | 46.8 ± 10.7 | 50.1 ± 8.7 |
| **Gender ratio, F/M** | 10/18 | 11/19 | --- | 20/20 | 19/21 |
| **HAM-D** | --- | 25.2 ± 6.1 | --- | --- | 24.5 ± 6.1. |
| **No. of previous episodes** | --- | 2.7 ± 3.1 | --- | --- | 2.1 ± 2.2 |
| **Non-REM-1, (%)** | **9.89 ±3.3** | **14 ± 6.6**$^{**}$ | **14.7 ± 7.8**$^{*}$ | **8.45 ± 4.7** | **13.9 ± 7.9**$^{***}$ |
| **Non-REM-2, (%)** | 48.7 ± 8.4 | 49.7 ± 11.9 | 49.0 ± 9.3 | 46.2 ± 9.0 | 50.3 ± 11.6 |

| | | | | | |
|---|---|---|---|---|---|
| **Non-REM-3, (%)** | 15.4 ± 7.5 | 13.4 ± 9.3 | 13.9 ± 9.9 | 16.2 ±8.4 | 13.1 ± 10.4 |
| **REM sleep, (%)** | **18.3 ± 4.5** | **13.0 ± 7.8**** | **14.0 ± 7.9*** | **18.6 ± 5.2** | **14.3 ± 8.7**** |
| **WASO, (%)** | 7.27 ± 6.3 | 9.09 ± 6.2 | 7.28 ± 5.4 | 10.4 ± 7.6 | 7.8 ± 5.7 |
| **Total non-REM time, (%)** | 64.2 ± 7.0 | 63.1 ± 8.9 | 62.9 ± 9.4 | 62.3 ± 8.0 | 63.4 ± 11.8 |
| **Total sleep time, min** | 467 ± 19.4 | 464 ± 4.4 | 466 ± 13.4 | 461 ± 27.1 | 462 ± 22.8 |
| **Sleep onset, min** | **26.3 ± 7.4** | **32.4 ± 14.1*** | 30.9 ± 14.3 | 22.7 ± 16.9 | 21.7 ± 13.4 |
| **Non-REM-3 onset, min** | 19 ± 12.4 | 24.1 ± 19.4 | 19.7 ± 11.6 | 21.5 ± 19.0 | 31.6 ± 30.6 |
| **REM onset, min** | **79.2 ± 31.3** | **186.0± 109.0***** | **173.0 ± 103.0***** | **74.0 ± 32.1** | **177.0 ± 110.7***** |

*REM – rapid eye movement sleep, WASO – wake after sleep onset, HAM-D – Hamilton Depression Rating Scale. Sleep stage percentages are given with respect to total sleep time. Note that non-REM sleep was defined as the combination of non-REM-2 and non-REM-3 without non-REM-1 sleep. Bold font indicates statistically significant differences between controls and medicated patients (*: p < .05; **: p < .01; ***: p < .001).*

Correlations between aperiodic slopes and sleep architecture are reported in Table S5.2. The following finding was observed in both Main Dataset and Replication Dataset 1: in 7-day medicated patients, flatter aperiodic slopes during non-REM sleep were linked to the lower proportion and delayed onset of REM sleep. In long-term medicated patients from Replication Dataset 2, no correlations between aperiodic slopes and sleep architecture were observed.

**Table S5.2: Correlations between aperiodic slopes and sleep stages proportions** (r)

| Dataset | Main study | | | Replication dataset 1 | | |
|---|---|---|---|---|---|---|
| **Group** | **Unmed** | **7-day med** | **HC** | **7-day med** | **28-day med** | **HC** |
| **N1 slope – WASO%** | n.s. | n.s. | n.s. | 0.409 | n.s. | n.s. |
| **N1 slope – N1%** | n.s. | 0.347 | n.s. | n.s. | n.s. | n.s. |
| **N1 slope – REM%** | n.s. | n.s. | n.s. | -0.361 | n.s. | n.s. |
| **N1 slope – REM onset** | n.s. | n.s. | n.s. | 0.449 | n.s. | n.s. |
| **N1 slope – SWS onset** | 0.335 | 0.351 | n.s. | n.s. | n.s. | n.s. |
| **N2 slope – WASO%** | n.s. | n.s. | n.s. | 0.452 | 0.418 | n.s. |
| **N2 slope – N1%** | n.s. | 0.479 | n.s. | n.s. | n.s. | 0.363 |
| **N2 slope – REM%** | n.s. | **-0.548*** | n.s. | **-0.396*** | n.s. | n.s. |
| **N2 slope – REM onset** | n.s. | **0.391*** | n.s. | **0.469*** | n.s. | n.s. |
| **N2 slope – TST** | n.s. | -0.328 | n.s. | n.s. | n.s. | n.s. |

| | | | | | | |
|---|---|---|---|---|---|---|
| **N3 slope – WASO** | n.s. | n.s. | 0.328 | 0.563 | 0.620 | n.s. |
| **N3 slope – N1%** | n.s. | 0.455 | n.s. | n.s. | n.s. | 0.379 |
| **N3 slope – N3%** | n.s. | n.s. | n.s. | -0.384 | -0.367 | -0.346 |
| **N3 slope – REM%** | n.s. | -0.357 | -0.591 | n.s. | n.s. | n.s. |

*Pearson correlations coefficients between aperiodic slopes measured during a particular sleep stage and features of sleep architecture are presented for each group and dataset separately. Only the r's associated with the statistically significant p-values are presented, meaning that the rest of the possible combinations between aperiodic slopes and sleep architecture features were statistically non-significant,* ***bold font with **** *marks the findings replicated in two independent datasets. Sleep stage percentages were calculated with respect to TST, TST – total sleep time, REM – rapid eye movement sleep, N – non-rapid eye movement sleep, WASO – wakefulness after sleep onset, r – Pearson correlation coefficient.*

## S5.1. Replication dataset 1

The results are presented in Table S5.3 and Fig.S5.1. Both 7-day and 28-day medicated patients showed flatter broadband and high-band slopes during the N1, N2, N3, and REM stages in all areas with medium to very large effect sizes compared to controls. Broadband and high-band slopes of the wakefulness after sleep onset were comparable.

Both 7-day and 28-day medicated patients showed flatter low-band slopes during N3 sleep in all areas with large effect sizes compared to controls. Low-band slopes of the wakefulness after sleep onset, N1, N2, and REM epochs were comparable.

## Table S5.3: Slopes in Replication dataset 1

| Mean | | 7-day medicated MDD | | | | | 28-day medicated MDD | | | | | HC | | | | |
|---|---|---|---|---|---|---|---|---|---|---|---|---|---|---|---|---|
| Broadband | Area/ Stage | F | C | P | O | T | F | C | P | O | T | F | C | P | O | T |
| | Wake | -1.41 | -1.29 | -1.63 | -1.71 | -1.33 | -1.39 | -1.28 | -1.65 | -1.74 | -1.33 | -1.50 | -1.38 | -1.70 | -1.76 | -1.42 |
| | N1 | -1.97 | -1.86 | 2.00 | -2.12 | -1.93 | -2.00 | -1.90 | -2.03 | -2.15 | -1.97 | -2.13 | -2.02 | -2.13 | -2.24 | -2.12 |
| | N2 | -2.44 | -2.33 | -2.42 | -2.50 | -2.37 | -2.49 | -2.39 | -2.46 | -2.56 | -2.41 | -2.62 | -2.49 | -2.52 | -2.65 | -2.59 |
| | N3 | -2.76 | -2.64 | -2.70 | -2.78 | -2.68 | -2.78 | -2.67 | -2.65 | -2.81 | -2.69 | -3.00 | -2.87 | -2.87 | -3.00 | -2.98 |
| | REM | -2.01 | -1.89 | -1.98 | -2.09 | -2.05 | -2.01 | -1.89 | -1.97 | -2.09 | -2.02 | -2.10 | -2.01 | -2.13 | -2.24 | -2.17 |
| Low band | Area/ Stage | F | C | P | O | T | F | C | P | O | T | F | C | P | O | T |
| | Wake | -0.95 | -0.77 | -0.99 | -1.10 | -0.87 | -1.01 | -0.85 | -1.08 | -1.19 | -0.94 | -0.99 | -0.79 | -0.97 | -1.00 | -0.86 |
| | N1 | -1.41 | -1.25 | -1.37 | -1.54 | -1.43 | -1.44 | -1.29 | -1.40 | -1.58 | -1.45 | -1.52 | -1.35 | -1.46 | -1.62 | -1.52 |
| | N2 | -1.92 | -1.79 | -1.89 | -2.03 | -1.93 | -1.96 | -1.84 | -1.92 | -2.06 | -1.96 | -2.05 | -1.89 | -1.95 | -2.12 | -2.04 |
| | N3 | -2.37 | -2.24 | -2.32 | -2.41 | -2.37 | -2.34 | -2.23 | -2.22 | -2.39 | -2.33 | -2.62 | -2.47 | -2.50 | -2.62 | -2.61 |
| | REM | -1.61 | -1.41 | -1.46 | -1.56 | -1.56 | -1.59 | -1.39 | -1.44 | -1.55 | -1.53 | -1.62 | -1.44 | -1.56 | -1.64 | -1.60 |
| High band | Area/ Stage | F | C | P | O | T | F | C | P | O | T | F | C | P | O | T |
| | Wake | -2.76 | -2.82 | -3.28 | -3.30 | -2.61 | -2.68 | -2.68 | -3.15 | -3.27 | -2.58 | -3.01 | -3.08 | -3.59 | -3.69 | -2.90 |
| | N1 | -3.91 | -3.90 | -4.08 | -4.03 | -3.68 | -3.91 | -3.91 | -4.07 | -4.11 | -3.76 | -4.40 | -4.38 | -4.50 | -4.57 | -4.25 |
| | N2 | -3.97 | -3.91 | -4.05 | -4.05 | -3.78 | -3.94 | -3.92 | -4.01 | -4.07 | -3.78 | -4.43 | -4.33 | -4.38 | -4.55 | -4.32 |
| | N3 | -3.90 | -3.78 | -3.90 | -3.98 | -3.71 | -3.91 | -3.83 | -3.60 | -3.98 | -3.80 | -4.21 | -4.09 | -4.09 | -4.31 | -4.11 |
| | REM | -3.90 | -3.98 | -4.10 | -4.28 | -4.09 | -3.87 | -3.96 | -4.04 | -4.23 | -4.04 | -4.32 | -4.37 | -4.47 | -4.70 | -4.54 |
| **Effect size** | | **7-day medicated MDD-HC** | | | | | **28-day medicated MDD-HC** | | | | | **7-day medicated-28-day medicated MDD** | | | | |
| Broadband | Area/ Stage | F | C | P | O | T | F | C | P | O | T | F | C | P | O | T |
| | W | 0.32 | 0.24 | 0.24 | 0.22 | 0.30 | 0.32 | 0.27 | 0.16 | 0.07 | 0.27 | -0.05 | -0.04 | 0.10 | 0.19 | 0.01 |
| | N1 | **0.90** | **0.74** | **0.70** | **0.81** | **0.90** | **0.74** | **0.65** | **0.55** | **0.55** | **0.76** | 0.19 | 0.25 | 0.22 | **0.38** | 0.27 |

| | | | | | | | | | | | | | | | | |
|---|---|---|---|---|---|---|---|---|---|---|---|---|---|---|---|---|
| | N2 | **1.05** | **0.97** | **0.75** | **0.97** | **1.07** | **0.85** | **0.71** | 0.42 | **0.57** | **0.97** | 0.34 | **0.42** | 0.29 | **0.39** | **0.38** |
| | N3 | **1.24** | **1.42** | **1.12** | **1.31** | **1.32** | **1.15** | **1.28** | **0.94** | **1.00** | **1.30** | 0.14 | 0.21 | 0.14 | 0.26 | 0.05 |
| | REM | **0.75** | **0.74** | **0.95** | **0.92** | **0.78** | **0.75** | **0.74** | **0.94** | **0.88** | **0.91** | **-0.03** | -0.01 | -0.04 | 0.02 | -0.26 |
| Low band | Area/ Stage | F | C | P | O | T | F | C | P | O | T | F | C | P | O | T |
| | Wake | 0.13 | 0.07 | -0.06 | -0.33 | -0.02 | -0.01 | -0.08 | -0.31 | **-0.63** | -0.16 | 0.18 | 0.19 | 0.43 | **0.36** | 0.18 |
| | N1 | 0.48 | 0.41 | 0.35 | 0.32 | 0.41 | 0.39 | 0.28 | 0.25 | 0.20 | 0.35 | 0.17 | 0.23 | 0.16 | 0.21 | 0.12 |
| | N2 | **0.65** | 0.49 | 0.30 | 0.43 | **0.51** | 0.44 | 0.25 | 0.15 | 0.29 | 0.41 | **0.36** | **0.42** | 0.23 | 0.24 | 0.21 |
| | N3 | **1.15** | **1.08** | **0.88** | **1.00** | **1.14** | **1.19** | **1.15** | **1.01** | **1.08** | **1.34** | -0.12 | -0.11 | -0.12 | -0.08 | -0.30 |
| | REM | 0.06 | 0.15 | 0.42 | 0.34 | 0.15 | 0.14 | 0.23 | 0.49 | 0.38 | 0.29 | -0.13 | -0.19 | -0.14 | -0.10 | -0.36 |
| High band | Area/ Stage | F | C | P | O | T | F | C | P | O | T | F | C | P | O | T |
| | Wake | 0.33 | 0.30 | 0.41 | **0.55** | 0.38 | 0.46 | 0.50 | **0.60** | **0.63** | 0.48 | -0.21 | -0.26 | -0.27 | -0.16 | -0.16 |
| | N1 | **0.91** | **0.87** | **1.00** | **1.23** | **0.99** | **0.94** | **0.95** | **1.00** | **1.05** | **0.90** | **-0.13** | -0.07 | -0.12 | 0.08 | 0.04 |
| | N2 | **1.10** | **1.07** | **1.03** | **1.23** | **1.11** | **1.11** | **1.05** | **1.01** | **1.12** | **1.10** | **-0.20** | -0.06 | -0.19 | -0.02 | -0.16 |
| | N3 | **0.69** | **0.79** | **0.62** | **0.84** | **0.76** | **0.66** | **0.65** | **0.56** | **0.77** | **0.67** | **-0.07** | 0.04 | -0.07 | -0.09 | 0.02 |
| | REM | **1.04** | **0.97** | **1.07** | **1.35** | **1.20** | **1.13** | **1.04** | **1.14** | **1.30** | **1.13** | **-0.21** | -0.19 | -0.25 | -0.26 | -0.20 |

***Bold** font indicates statistically significant p-values after the correction for multiple comparisons, gray font indicates non-significant values, effect sizes are interpreted as small (0.2–0.5), medium (0.5–0.8), and large (0.8–1.2), MDD – major depressive disorder, HC – healthy controls, W – wake, F – frontal, C – central, P – parietal, O – occipital, T – temporal electrodes, REM – rapid eye movement sleep, N – non–rapid eye movement sleep.*

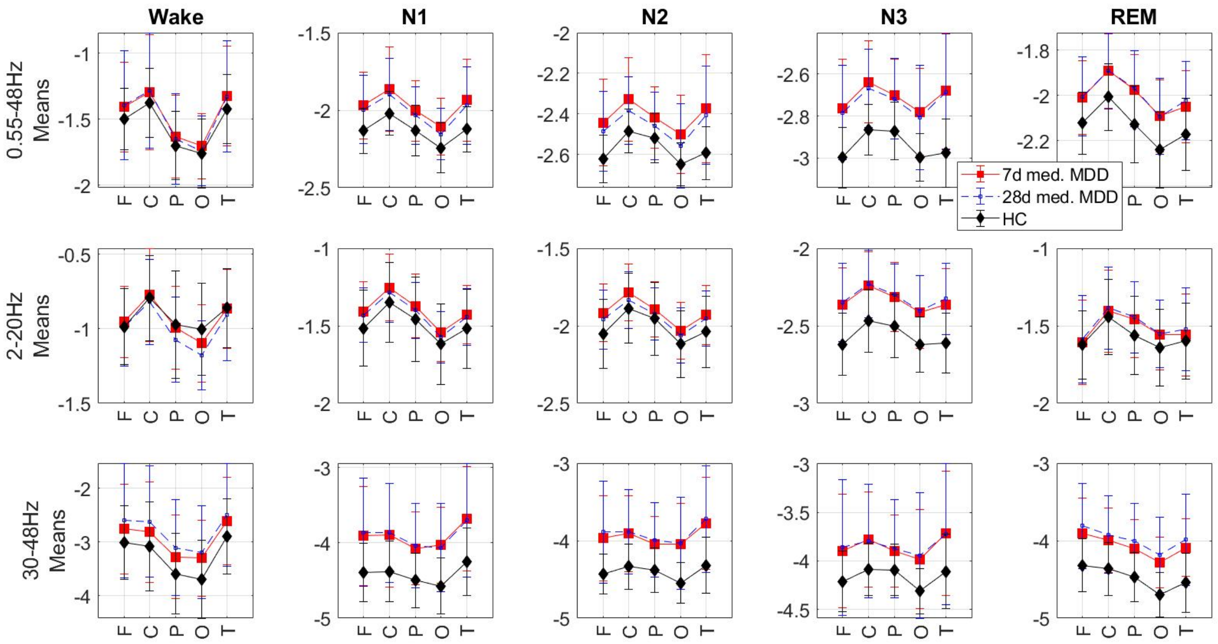


**Figure S5.1. Slopes in Replication dataset 1.** The slopes of the aperiodic power component in the 0.55-48 Hz **(top)**, 2-20 Hz **(middle)**, and 30-48 Hz **(bottom)** bands were averaged over each sleep stage over each area of interest. Patients at 7-day (red) and 28-day (blue) of medication treatment show flatter slopes (more positive values) of the high-band (top) and broadband (top) power than controls (black) in all areas during all sleep stages. For the low band (middle), the difference was prominent only during N3 sleep. MDD – 30 major depressive disorder, HC – 30 healthy controls, F – frontal, C – central, P – parietal, O – occipital, T – temporal electrodes.

## Medication effect

To replicate the finding on the specific effect of the antidepressant class observed in the Main Text, we stratified the 7-day medicated patients from Replication Dataset 1 by their medication class. The demographic and clinical characteristics of the subgroups of patients stratified by the medication class are reported in Table S5.4. In contrast to the Main dataset, we found that aperiodic activity was comparable in the patients who took REM-suppressive and REM non-suppressive antidepressants as well as in patients who took SNRIs and non-SNRIs or other than SNRIs REM-

suppressive antidepressants. The most probable explanation for this discrepancy is that in the main study, patients took different REM suppressive agents than in the replication study. It is well-known that different agents even from the same class can show opposite modes of action on sleep (7). Moreover, in clinical settings, response to antidepressants is highly variable (7). Other reasons for this discrepancy include the older age and higher number of depression episodes (that might correspond to the disorder progression) of the patients from the replication dataset. Likewise, the replication analysis had low statistical power and unbalanced groups (21 vs 9 patients in the replication dataset compared to 21 vs 17 patients in the main study).

At the same time, in the Replication Dataset 1, the patients who took TCA showed flatter aperiodic slopes during REM sleep in the central, parietal, occipital, and temporal areas with mediume to large effect sizes compared to their pooled controls (all p-values ranging from 0.011 to 0.025, all Cohen's d-values ranging from 0.71 to 0.97). However, this finding did not pass the correction for multiple comparisons (25 non-parametric tests for 5 stages by 5 areas separately, Table S5.5).

**Table S5.4: Demographic and clinical characteristics of the subgroups of patients by medication class** (mean ± SD)

| Medication class | Sample size | Age | Gender | No. of previous episodes | HAM-D baseline | HAM-D 7-day | HAM-D 28-day |
|---|---|---|---|---|---|---|---|
| TCA (trimipramine, amitriptyline, amitriptylinoxide, doxepine, clomipramine, nortriptyline) | 10 | 47.5±11.5 | 6 F | 3.7±3.7 | 24.6±6.2 | 17.9±6.3 | 15.16.2 |
| SNRI (venlafaxine, duloxetine, sertralin, citalopram) | 12 | 47.6±19.9 | 7 F | 2.4±2.2 | 23.9±6.4 | 20.8±7.2 | 14.3±9.6 |
| REM suppressive (SNRI, reboxetine, amitriptylinoxide, doxepine, clomipramine, nortriptyline) | 21 | 47.1±16.8 | 16 F | 3.2±3.1 | 24.3±6.2 | 19.0±7.4 | 13.5±8.1 |
| REM non-suppressive (mirtazapine, bupropione, trimipramine) | 9 | 44.4±17.7 | 5 F | 2.4±3.2 | 26.6±5.8 | 21.9±5.2 | 15.2±8.5 |

*The NARI (reboxetine, n=1), SSRI (n=2), and NDRI (n=3) subgroups were not analyzed separately due to small sample size. HAM-D – Hamilton Depression Rating Scale, REM – rapid eye movement sleep, SD – standard deviation, NaSSA – noradrenergic and specific serotonergic antidepressants, NDRI – norepinephrine-dopamine reuptake inhibitor, SNRI – serotonin-norepinephrine reuptake inhibitors, SSRI – selective serotonin reuptake inhibitors, TCA – tricyclic antidepressants.*

## Table S5.5: Effect of antidepressants on aperiodic activity

| Main study | | | Replication study 1 | | |
|---|---|---|---|---|---|
| **Subgroups** | | **Slope difference** | **Subgroups** | | **Slope difference** |
| 13 SSRI | 25 non-SSRI | n.s. | 2 SSRI | 28 non-SSRI | NA |
| 8 TCA | 30 non-TCA | n.s. | 10 TCA | 20 non-TCA | ↑ REM C, P, O, T |
| 6 NDRI | 32 non-NDRI | n.s. | 3 NDRI | 27 non-NDRI | NA |
| 6 SNRI | 32 non-SNRI | ↑ N1, **N2, N3, REM** F, C, P, O, T | 12 SNRI | 18 non-SNRI | n.s. |
| 21 REM suppressive | 17 REM non-suppressive | ↑ **N1, N2, N3, REM** F, C, P, O, T | 21 REM suppressive | 9 REM non-suppressive | n.s. |
| 6 SNRI (REM suppressive) | 15 REM suppressive non-SNRI | ↑ REM F, C, P, O, T | 12 SNRI | 18 REM non-suppressive | n.s. |
| 6 SNRI (REM suppressive) | 13 SSRI (REM suppressive) | ↑ REM F, C, P, O, T | 12 SNRI | 2 SSRI | NA |

***Bold*** *font indicates the finding that remained statistically significant after the correction for multiple comparisons, F – frontal, C – central, P – parietal, O – occipital, T – temporal electrodes, n.s. – non-significant, REM – rapid eye movement sleep, N – non–rapid eye movement sleep, NaSSA – noradrenergic and specific serotonergic antidepressants, NDRI – norepinephrine-dopamine reuptake inhibitor, SNRI – serotonin-norepinephrine reuptake inhibitors, SSRI – selective serotonin reuptake inhibitors, TCA – tricyclic antidepressants*

**S5.2. Replication dataset 2**

The results are presented in Table S5.6 and Fig.S5.2. The long-term medicated patients showed flatter broadband and low-band aperiodic slopes during N1, N2, N3, and REM sleep (all p-values<0.003) in the central electrodes (other channels were unavailable in this dataset) with medium effect sizes compared to controls.

The patients showed flatter high-band aperiodic slopes during N2, N3, and REM sleep (all p-values<0.003) in the central electrodes with medium effect sizes compared to controls. Broadband, low, and high-band slopes of the wakefulness after sleep onset were comparable.

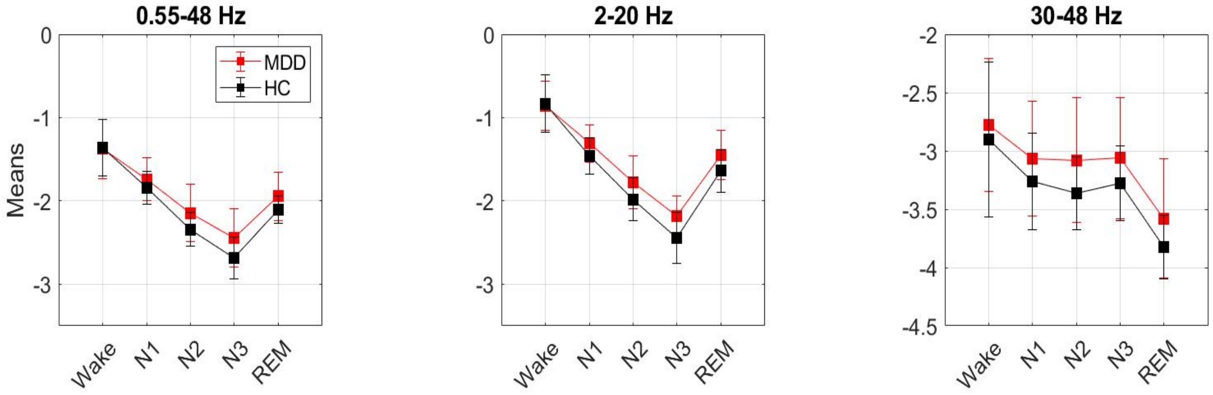


**Figure S5.2. Aperiodic slopes in Replication dataset 2.** The slopes of the aperiodic power component in the 0.55-48Hz (**left**), 2-20Hz (**middle**), and 30-48Hz (**right**) bands were averaged over each sleep stage over the central electrodes. Long-term medicated patients (red) show flatter slopes (more positive values) during non-REM and REM sleep in all frequency bands compared to controls (black). MDD – 40 long-term medicated major depressive disorder patients, HC – 40 healthy controls.

**Table S5.6: Slopes in Replication dataset 2**

| Groups | | Long-term medicated MDD | Controls | MDD-Controls |
|---|---|---|---|---|
| Band | Stage | Slope means | | Effect size |
| Broadband | Wake | -1.38 | -1.36 | -0.05 |
| | N1 | -1.74 | -1.84 | **0.45** |
| | N2 | -2.15 | -2.34 | **0.70** |
| | N3 | -2.44 | -2.68 | **0.79** |
| | REM | -1.95 | -2.11 | **0.69** |
| Low band | Wake | -0.86 | -0.83 | -0.09 |
| | N1 | -1.31 | -1.46 | **0.69** |
| | N2 | -1.78 | -1.98 | **0.71** |
| | N3 | -2.18 | -2.44 | **0.95** |
| | REM | -1.45 | -1.64 | **0.70** |
| High band | Wake | -2.78 | -2.90 | 0.20 |
| | N1 | -3.07 | -3.26 | 0.43 |
| | N2 | -3.08 | -3.37 | **0.66** |
| | N3 | -3.06 | -3.28 | **0.50** |
| | REM | -3.58 | -3.83 | **0.60** |

***Bold** font indicates statistically significant p-values, gray font indicates non-significant values, effect sizes are interpreted as small (0.2–0.5), medium (0.5–0.8), and large (0.8–1.2), all values were averaged over the central electrodes, MDD – major depressive disorder, REM – rapid eye movement sleep, N – non–rapid eye movement sleep.*

### S5.3. Comparison between the datasets

The comparison between the Main dataset, Replication dataset 1, and Replication Dataset 2 are presented in Fig. S5.3 and Table S5.7. We found that medicated patients from all datasets showed flatter slopes during both REM and non-REM sleep.

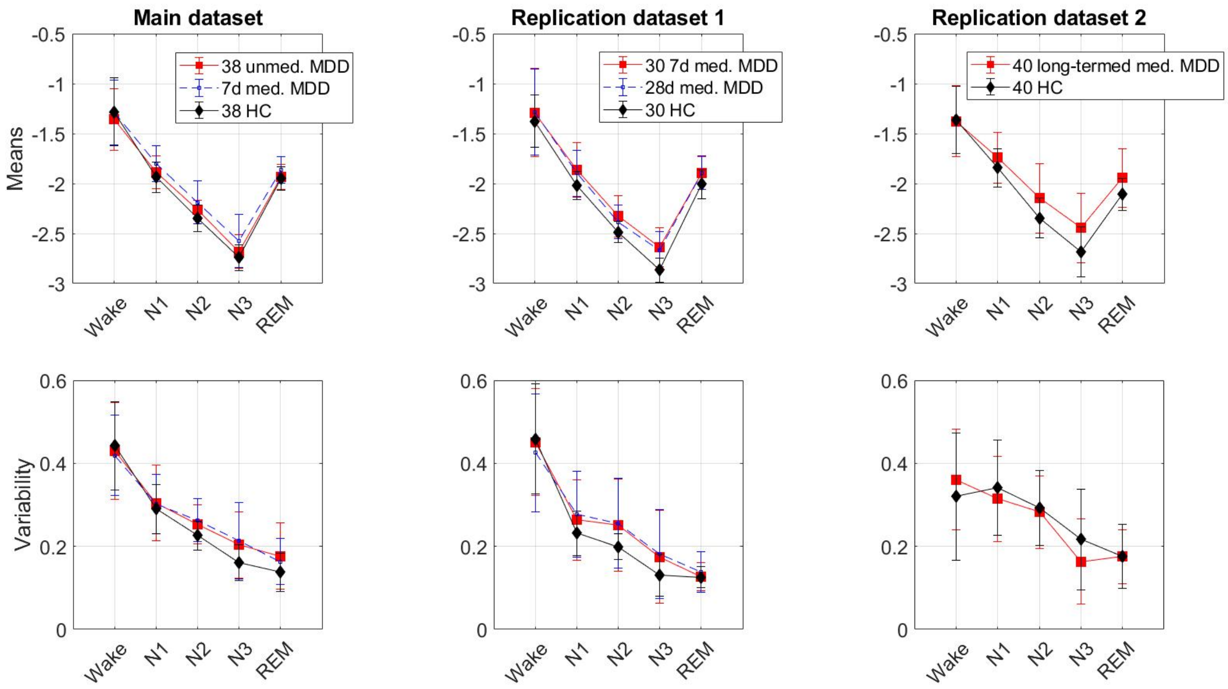


**Figure S5.3. Main and replication datasets comparison.** The slopes of the aperiodic broadband power component were averaged over each sleep stage over the central electrodes. Medicated patients from all datasets show flatter slopes during both REM and non-REM sleep compared to controls. MDD – major depressive disorder patients, HC – healthy controls.

## Table S5.7: Summary of the *post hoc* findings

| Band | Broadband | | Low band | | High band | |
|---|---|---|---|---|---|---|
| **Main dataset** | | | | | | |
| **State/ Stage** | **Unmed.** | **7-day med.** | **Unmed.** | **7-day med.** | **Unmed.** | **7-day med.** |
| Wake | n.s. | n.s. | n.s. | n.s. | n.s. | n.s. |
| N1 | n.s. | ↑ F, C, P, O, T | n.s. | ↑ C, P, T | n.s. | ↑ F, C, O |
| N2 | ↑ F, C, P, O, T | ↑ F, C, P, O, T | ↑ F, C, P, O, T | ↑ F, C, P, O, T | n.s. | ↑ F, C, O |
| N3 | ↑ F, T | ↑ F, C, P, O, T | ↑ F, C, P, O, T | ↑ F, C, P, O, T | n.s. | ↑ F, C, T |
| REM | n.s. | ↑ F, C, P, O, T | n.s. | ↑ F, C, P, O, T | n.s. | n.s. |
| **Replication dataset 1** | | | | | | |
| **State/ Stage** | **7-day med.** | **28-day med.** | **7-day med.** | **28-day med.** | **7-day med.** | **28-day med.** |
| Wake | n.s. | n.s. | n.s. | ↑ O | ↑ O | ↑ P, O |
| N1 | ↑ F, C, P, O, T | ↑ F, C, P, O, T | n.s. | n.s. | ↑ F, C, P, O, T | ↑ F, C, P, O, T |
| N2 | ↑ F, C, P, O, T | ↑ F, C, O, T | ↑ F, T | n.s. | ↑ F, C, P, O, T | ↑ F, C, P, O, T |
| N3 | ↑ F, C, P, O, T | ↑ F, C, P, O, T | ↑ F, C, P, O, T | ↑ F, C, P, O, T | ↑ F, C, P, O, T | ↑ F, C, P, O, T |
| REM | ↑ F, C, P, O, T | ↑ F, C, P, O, T | n.s. | n.s. | ↑ F, C, P, O, T | ↑ F, C, P, O, T |
| **Replication dataset 2** | | | | | | |
| **State/ Stage** | **Long-term medicated** | | **Long-term medicated** | | **Long-term medicated.** | |
| Wake | n.s. | | n.s. | | n.s. | |
| N1 | ↑ | | ↑ | | n.s. | |
| N2 | ↑ | | ↑ | | ↑ | |
| N3 | ↑ | | ↑ | | ↑ | |
| REM | ↑ | | ↑ | | ↑ | |

*Only central electrodes were available for Replication dataset 2, F – frontal, C – central, P – parietal, O – occipital, T – temporal electrodes, n.s. – non-significant, unmed. – unmedicated, med. – medicated, ↑ – increased compared to controls, REM – rapid eye movement sleep, N – non–rapid eye movement sleep.*

# Supplementary Material 6

**Table S6.1: Percentage of excluded epochs**

| **Epochs** | **All epochs** | | | **Non-REM epochs** | | |
|---|---|---|---|---|---|---|
| **Dataset** | **Controls** | **Patients (record 1)** | **Patients (record 2)** | **Controls** | **Patients (record 1)** | **Patients (record 2)** |
| **Main dataset** | 6.90% | 7.40% | 9.50% | 5.00% | 5.80% | 7.50% |
| **Replication Dataset 1** | 3.25% | 4.10% | 4.50% | 2.20% | 3.40% | 3.10% |
| **Replication Dataset 2** | 7.00% | 7.50% | --- | 3.60% | 3.90% | --- |

**Supplementary References**


1 Wen H, Liu Z. Separating fractal and oscillatory components in the power spectrum of neurophysiological signal. *Brain topography.* 2016 Jan;29(1):13-26.

2 Gao R, Peterson EJ, Voytek B. Inferring synaptic excitation/inhibition balance from field potentials. *Neuroimage.* 2017;158: 70–78. https://doi.org/10.1016/j.neuroimage.2017.06.078

3 Lendner JD, Helfrich RF, Mander BA, Romundstad L, Lin JJ, Walker MP, et al. An electrophysiological marker of arousal level in humans. *Elife.* 2020 Jul 28;9:e55092. https://doi.org/10.7554/eLife.55092

4 Kozhemiako N, Mylonas D, Pan JQ, Prerau MJ, Redline S, Purcell SM. Sources of variation in the spectral slope of the sleep EEG. bioRxiv. 2021 Jan 1 https://doi.org/10.1101/2021.11.08.467763

5 Miskovic V, MacDonald KJ, Rhodes LJ, Cote KA. Changes in EEG multiscale entropy and power-law frequency scaling during the human sleep cycle. *Human Brain Mapp.* 2019 Feb 1;40(2):538-51. https://doi.org/10.1002/hbm.24393

6 Bovy L, Weber FD, Tendolkar I, Fernández G, Czisch M, Steiger A, et al. Non-REM sleep in major depressive disorder. *bioRxiv.* 2021 Jan 1. https://doi.org/10.1101/2021.03.19.436132

7 Wichniak A, Wierzbicka A, Walęcka M, Jernajczyk W. Effects of antidepressants on sleep. *Current psychiatry reports.* 2017 Sep;19(9):1-7. https://doi.org/10.1007/s11920-017-0816-4
